# Acquisition of virulence factors in livestock-associated MRSA: Lysogenic conversion of CC398 strains by virulence gene-containing phages

**DOI:** 10.1038/s41598-017-02175-4

**Published:** 2017-05-17

**Authors:** Britta Kraushaar, Jens Andre Hammerl, Marina Kienöl, Marie Luise Heinig, Nina Sperling, Mai Dinh Thanh, Jochen Reetz, Claudia Jäckel, Alexandra Fetsch, Stefan Hertwig

**Affiliations:** German Federal Institute for Risk Assessment, Department Biological Safety, Berlin, Germany

## Abstract

*Staphylococcus aureus* MRSA strains belonging to the clonal complex 398 (CC398) are highly prevalent in livestock and companion animals but may also cause serious infections in humans. CC398 strains in livestock usually do not possess well-known virulence factors that can be frequently found in other MRSA sequence types (ST). Since many staphylococcal virulence genes are residing on the genomes of temperate phages, the question arises why livestock-associated (LA-) CC398 strains are only rarely infected by those phages. We isolated and characterized four temperate phages (P240, P282, P630 and P1105) containing genes of the immune evasion cluster (IEC) and/or for the Panton-Valentine leucocidin (PVL). Sequence analysis of the phage genomes showed that they are closely related to known phages and that the DNA region encoding lysis proteins, virulence factors and the integrase exhibits numerous DNA repeats which may facilitate genomic rearrangements. All phages lysed and lysogenized LA-CC398 strains. Integration of IEC phage P282 was detected at ten sites of the hosts’ chromosome. The prophages were stably inherited in LA-CC398 and enterotoxin A, staphylokinase and PVL toxin were produced. The data demonstrate that lysogenic conversion of LA-CC398 strains by virulence-associated phages may occur and that new pathotypes may emerge by this mechanism.

## Introduction

Methicillin-resistant *Staphylococcus* (*S*.) *aureus* (MRSA) is an important human pathogen not only in health care systems but also in community settings^1^. In the last decade, MRSA has also emerged in livestock^2^. The predominant MRSA lineage detected in animals in Europe and USA is of clonal complex (CC) 398 comprising a heterogeneous group of strains with currently 43 sequence types (ST) and a variety of *spa* types^3^. *Staphylococcus aureus* CC398 strains cluster into two distinct phylogenetic clades, a livestock clade and a basal human clade^4^. Livestock-associated (LA-) CC398 strains may cause diseases in both humans and animals, e.g. wound infections and bacteremia^5^, foot joint infections in turkeys^6^ and bovine mastitis^7, 8^. CC398 strains are often multiresistant to several classes of antimicrobial agents but they usually lack well-known virulence genes^9^, of which a significant number is located on mobile genetic elements, e.g. temperate phages^10^. However, there are distinct differences between LA- and human-adapted CC398 strains. Whereas strains belonging to the human clade frequently harbor ΦSa3, a ß-hemolysin (*hlb*) converting integrase (*int*) group 3 prophage that carries the human-specific immune evasion cluster IEC^11^, this prophage is usually absent in LA-CC398 strains. IEC encodes several immune modulating proteins which counteract innate immunity. The most common proteins are the staphylokinase (SAK), staphylococcal complement inhibitor (SCIN) and chemotaxis inhibitory protein (CHIPS), but some IEC clusters also contain genes for staphylococcal enterotoxins like enterotoxin A (SEA) or enterotoxin P (SEP)^12^. SEA belongs to the classical staphylococcal enterotoxins and exhibits emetic and superantigenic activity^13^. Enterotoxin-producing *S. aureus* is one of the leading causes of foodborne poisoning^14^ but the strains are often methicillin-sensitive. The bi-component exotoxin Panton-Valentine leucocidin (PVL) encoded by *int* group 2 phages is common in community-acquired (CA-) MRSA^15^, but has only occasionally been found in CC398^16^. Its contribution to the virulence of MRSA is not fully understood, though it destroys polymorphonuclear granulocytes by forming pores, which leads to lysis of the cells^17^. It is still unclear why virulence gene containing phages have to date only rarely been detected in LA-MRSA CC398 strains. One reason could be an insusceptibility of LA-CC398 strains to those phages. The strains may lack a receptor for phage adsorption or a suitable integration site within their chromosome. They could also possess defence systems that can degrade phage DNA. Van der Mee-Marquet *et al*.^18^ studied a ß-hemolysin-converting ΦSa3 phage in a LA-MRSA CC398 strain and found that the prophage was not stable and needed a helper phage for infection. This finding suggests that LA-MRSA CC398 strains might not be suitable hosts for virulence gene-containing phages. On the other hand, many LA-CC398 strains are lysogenic and harbor one or even several prophages^19, 20^. Most of the identified prophages belong to the *int* groups 2 and 6, but known virulence genes have not been detected on their genomes. Nevertheless, the fact that such prophages occur in LA-CC398 strains indicates that basic requirements for a phage infection are fulfilled.

In this work, we isolated and characterized four temperate *S. aureus* phages containing a different composition of virulence genes and analysed their genomes. Bioinformatic analysis revealed repetitive DNA sequences on the genomes probably associated with recombinational events. All phages were able to infect and to lysogenize LA-CC398 strains. We determined multiple integration sites of the ΦSa3 phage P282 within the *S. aureus* chromosome and show that the phage-encoded virulence factors are expressed in LA-CC398, which may increase the virulence of these strains *in vivo*.

## Results

### Isolation of the virulence gene*-*carrying *S. aureus* phages P240, P282, P630 and P1105

**Figure 1:**
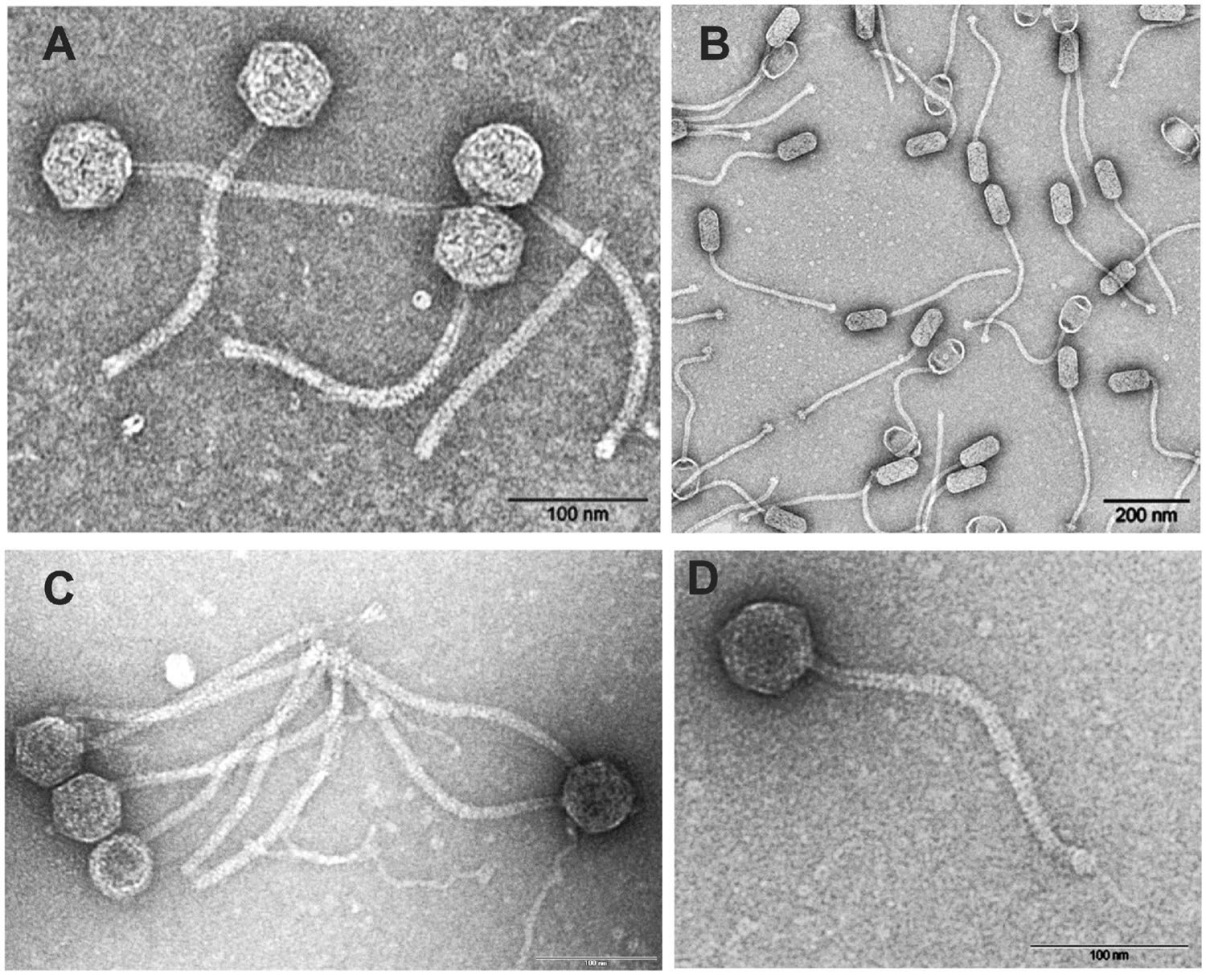
Electron micrographs of the phages P1105 (**A**), P240 (**B**), P630 (**C**) and P282 (**D**).

To identify virulence gene-containing phages in *S. aureus* that should be used in subsequent experiments with LA-MRSA CC398, a large number of strains belonging to various MLST types was analysed by microarrays for the presence of virulence genes known to be located on the genomes of temperate phages^10^. Four strains (10S00630, 11S00282, 11S01105 and 13-ST00240) isolated from human clinical samples and from poultry harbored genes of the IEC cluster and/or encoded PVL (Table 1). To examine whether active prophages reside in these strains, mitomycin C induction was performed. In all prepared lysates the respective virulence genes were detected by PCR (data not shown). To identify suitable hosts for phage propagation, the lysates were spotted on a set of LA-MRSA CC398 strains. For each lysate at least one susceptible indicator strain was found. As it was uncertain how many prophages had been induced in each strain, single plaques were analysed by PCR targeting the respective virulence genes, which resulted in the isolation of four phages containing a different composition of these genes (Table 1). Using multiplex-PCR (see Materials and Methods) the *int* group of the phages was determined. Two phages (P240 and P1105) belong to *int* group 2. Both phages possess *pvl* genes, but P1105 additionally carries the enterotoxin A gene *sea*. Phage P282 is a member of *int* group 3 (ΦSa3) and contains three IEC genes (*chp*, *sak*, *scn*) whereas *int* group 6 phage P630 carries the virulence gene *sea*. All phages are members of the *Siphoviridae* family. Though, while P282, P630 and P1105 possess an isometric head, the head of P240 is prolate (Fig. 1).

**Table 1:** Origin and some characteristics of the virulence gene containing phages used in this study.

Strain		Isolated from	*Spa* type	MLST type	Phage	Integrase type	Virulence genes
10S00630	MRSA	turkey, nasal swab	t002	ST1791	P630	Sa6	*sea*
11S00282	MRSA	chicken, meat	t899	ST398	P282	Sa3	*chp*, *sak*, *scn*
11S01105	MRSA	human, abscess	t657	ST772	P1105	Sa2	*lukF-PVL*, *lukS-PVL*, *sea*
13-ST00240	MSSA	human, wound swab	t034	ST398	P240	Sa2	*lukF-PVL*, *lukS-PVL*

### Sequence analyses of the phages disclosed repetitive DNA sequences probably involved in genomic rearrangements

We determined the sequences of the four phages and aligned their genomes using the start codon of the terminase small subunit gene as starting point. The phages P282, P630 and P1105 have similar genome sizes between 40.5 kb and 42.5 kb, the genome of P240 is a little larger (46 kb). All phages showed significant homologies to previously described *S. aureus* phages (Supplementary Material Table S2). *Int* 2 phage P240 is a typical member of the prolate-headed group 2 of Sfi21-like PVL phages like phiSLT^21^, which is 99% identical over 72% of the genome. According to the current taxonomy of staphylococcal phages, this group belongs to the genus “3alikevirus”^22^. The closest relatives of *int* 3 phage P282 are other ΦSa3 phages, e.g. phiNM3^23^ that is 98% identical over 69% of the genome. *Int* 6 phage P630 is similarly related to ΦSa3 phages, the strongest overall homologies, however, exist to group 3 PVL phage phi7247PVL^24^. Finally *int* group 2 phage P1105 isolated from a ST772 strain is nearly identical (99% identical over 99% of the genome) to the ST772 phages ΦIND772PVL and phiSa119 isolated in India and Italy^25, 26^, both encoding PVL and enterotoxin A. As shown in Figure S1 the isometric-headed phages P282, P630 and P1105 are much more closely related to each other than to P240 and can be allocated to the genus “77likevirus”^22^, the type phage of which is phage 77^27^. Particularly the left arm of the genomes up to the holin gene, which mainly encodes proteins for DNA packaging and virion assembly are very similar. The large terminase subunits of the three phages are even identical and differ by only one amino acid from the terminase of phage 77. Though, only little homologies exist to terminases of other members of the genus “77likevirus”, e.g. phiPVL108, which is a group 1 Sfi21-like *cos*-site PVL phage^28^. We therefore analysed restriction digests of phage P282 and P240 by standard procedures (heating and cooling, analysis of ligated DNA). While 3′-overhangs of ten nucleotides (GGCGGGGGCC) were easily detectable in the P240 genome, which is in accordance with other group 2 Sfi21-like PVL phages^29, 30^ we did not identify any *cos*-site in P282. Treatment of P282 DNA with Bal31 prior to digestion with restriction endonucleases resulted in a degradation of all restriction fragments (data not shown). We therefore speculate that the P282 genome is circularly permuted. A sequence (5′-CAGATTTATCTC-3′) very similar to the P22 consensus *pac*-site (5′-AAGATTTATCTG-3′) exists on the P282 genome, albeit far away from the gene for the small terminase subunit. Nevertheless, our data rather suggest headful packaging of P282 DNA than packaging of molecules with cohesive ends. Hence, the genus “77likevirus” may be comprised of both *cos-* and *pac*-site phages.

As with the terminase, structural proteins (major capsid protein, tail protein) of P282, P630 and P1105 are highly homologous to each other and to structural proteins of phage 77, while the corresponding proteins of group 1 (e.g. phiPVL108) and group 2 (e.g. phiSLT) PVL phages significantly diverge. Thus, DNA packaging and virus assembly obviously occur in a similar way in P282, P630 and P1105. It is recalled that these phages possess different virulence and integrase genes. On the other hand, P240, which contains PVL genes like P1105 and belongs to the same *int* group is only distantly related to the other phages.

**Figure 2:**
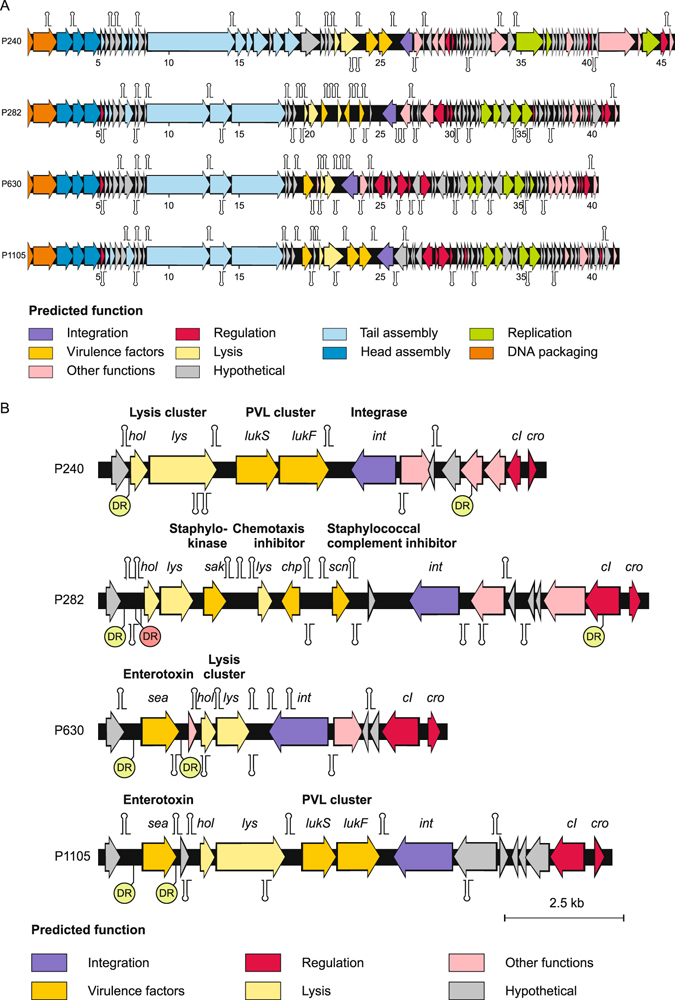
Genetic maps of P240, P282, P630 and P1105. (**A**) Whole genome comparison. Putative genes are coloured according to the predicted functions of their products. The position of putative Rho-independent transcription terminators are indicated. (**B**) Arrangement of virulence genes and genes for cell lysis, the integrase and repressors proteins. The positions of perfect (green) and imperfect (red) direct repeats are indicated.

Compared to the left arm, the right arm of the phage genomes is much more diverse. It mainly contains genes coding for lysis proteins, virulence factors, the integrase, repressors and replication proteins (Fig. 2A). This DNA region is apparently prone to rearrangements leading to new compositions of genes. El Haddad and Moineau^31^ studied a deletion mutant of PVL phage LH1 and identified a heptanucleotide direct repeat (5′-TTTTACA-3′) and a palindrome (5′-ATTTAGTACNNNGTACTAAAT-3′) that possibly caused the deletion of *pvl* and *int* genes. The palindrome is also present in other staphylococcal siphophages where it is situated adjacent to *int* and might have an important regulatory function^32^. We analysed the P630 and P1105 genome in detail since these two phages possess uncommon combinations of genes, namely *pvl*/*sea* (P1105) and *sea*/*int* 6 (P630). Altogether, 14 copies of the LH1 direct repeat were identified on the genomes of these phages. In P630 one direct repeat overlaps the start codon of the group 6 *int* gene. Moreover, the palindromic sequence mentioned above was found 60 bp upstream of the direct repeat. Thus, *sea* phage P630 may have acquired the *int* 6 gene by recombination with another phage.

**Figure 3:**
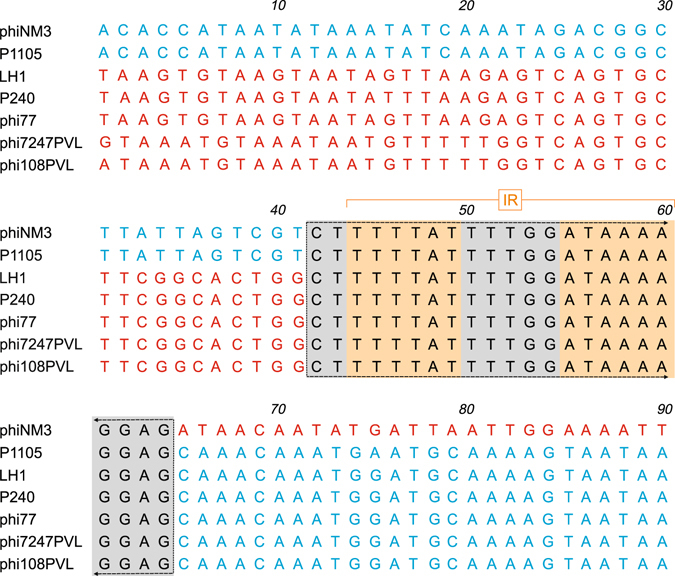
The transition point between homologous and non-homologous DNA segments of P1105 and other phages reveals striking consistency. The inverted repeat present in all phage genomes is shown in orange and grey. It separates sequences, which are identical in P1105 and phiNM3 from sequences that are identical in P1105 and the other phages. Homologous sequences are depicted in blue.

In P630 and P1105, the *sea* enterotoxin A gene is located to the left of the holin gene (Fig. 2B). In both phages, *sea* is flanked by a direct repeat (5′-CTTTTTATTTTG-3′). This sequence occurs in many phages where it is similarly located upstream of the holin gene and may play a role in recombination events. The comparison of P1105 with ΦSa3 phage phiNM3 and group 2 PVL phages (LH1, P240) showed that the left arm of the P1105 genome up to position 20,775 is closely related to phiNM3 while upstream sequences share significant homology with the PVL phages (Fig. S2). The analysis of the transition point revealed the sequence 5′-CTTTTTATTTTG-3′ in all four genomes. Moreover, at this position the consensus sequence is expanded by further identical nucleotides creating an inverted repeat, which may form a stem-loop structure (Fig. 3). The same sequence was found in other phages (e.g. 77, phi108PVL, phi7247PVL) where it links sequences that are homologous respectively non-homologous to P1105. We also analysed the ends of a homologous DNA stretch in P630 and phi7247PVL and found the consensus sequence to be located there. Van der Mee-Marquet *et al*.^18^ reported on the recombination of the ΦSa3 prophage StauST398-4pro with a defective ΦMR11 related helper prophage (StauST398-5pro) resulting in a phage (StauST398-1) that had lost part of the IEC cluster. Our analyses showed that both prophages, which share only little overall DNA homology, contain the extended consensus sequence (the first nucleotide C is missing in the helper prophage) upstream of the holin gene, close to the recombination site. These all are clear signs that the identified sequences are involved in both the aquisitition of new genes and the recombination of whole phage genomes.

### A large number of LA-MRSA CC398 strains exhibit sensitivity to the virulence gene-containing phages

In this study we first determined the range of MRSA CC398 strains which is susceptible to the four phages. Seventy-two MRSA CC398 strains representing 44 *spa* types mainly isolated from different stages of the swine, cattle and poultry food chain were tested (Supplementary Material, Table S1). Lytic activity was observed with all phages (Table 2). Phage P282 lysed 44% of the strains. The other phages infected a lower number of strains. Some strains were lysed by all phages. We did not detect any specificity for certain *spa* types since eighteen *spa* types were infected by at least one phage. In the next step the potential of the phages to lysogenize LA-MRSA CC398 strains was investigated. Susceptible strains were infected with the phages and subsequently analysed with respect to their prophage content. All phages were able to lysogenize LA-MRSA CC398. Prophages were detected in the lysogens by PCR using primers targeting the respective *int* and virulence genes. Moreover, upon mitomycin C treatment, the virulence gene-containing phages were released from the lysogenized MRSA CC398 strains proved by plaque assays and electron microscopy. As two of the indicator strains (10S01355 and 13-ST00030) do not possess an inherent prophage, only the phages P240, P282 or P630 (Fig. 1) were visible in lysates of the lysogenized derivatives. By contrast, indicator strain 10S00170 possesses an own *int* group 6 prophage. All particles released from this strain exhibited an elongated head while lysates of its derivative lysogenized by P1105 showed two different phage particles, one with an elongated and one with an isometric head (data not shown).

**Table 2:** Lytic activity of the phages on livestock-associated CC398 strains.

	P1105	P240	P630	P282
**Origin of the strains**				
cattle (dust sample, n = 1)	0	1	0	1
cattle (nasal swab, n = 10)	3	5	1	8
cattle (carcass, n = 8)	1	1	1	3
chicken (dust sample, n = 1)	0	1	1	1
chicken (skin swab, n = 1)	0	0	1	1
chicken (meat, n = 2)	0	2	0	1
swine (dust sample, n = 5)	1	1	1	2
swine (boot swab, n = 1)	0	0	0	0
swine (nasal swab, n = 16)	1	1	0	4
swine (meat, n = 3)	0	0	1	0
swine (organs, n = 1)	0	0	0	0
turkey (dust sample, n = 7)	0	1	0	3
turkey (skin swab, n = 7)	0	1	1	2
turkey (tracheal swab, n = 1)	0	0	0	0
turkey (meat, n = 5)	1	3	3	4
minced meat (n = 2)	0	0	0	1
rabbit (organs, n = 1)	0	0	0	0
***spa*** **types**				
t011 (n = 5)	0	2	0	2
t034 (n = 10)	1	0	3	6
t108 (n = 2)	0	0	0	0
t899 (n = 1)	0	1	0	1
t1456 (n = 1)	0	1	0	1
t2346 (n = 14)	4	7	1	10
Others (n = 39*)	2	6	6	11
**Total numbers (n** = **72)**	**7**	**17**	**10**	**31**

The numbers of sensitive strains and the numbers of tested strains (in parentheses) are given. *Detailed information on suceptible *spa* types is given in Supplemental Material Table S1.

### ΦSa3 phage P282 integrates at numerous sites of the CC398 chromosome

ß-hemolysin converting phages are widespread in *S. aureus* but have only rarely been found in LA-MRSA CC398 strains, the reason for this finding has not been elucidated yet. We therefore studied the lysogenic properties of P282 in more detail. As lysogenization might be hampered by the presence of endogenous phages, we first determined the prophage content of 32 MRSA CC398 strains that were susceptible to P282 lytic infection. The multiplex PCR analysis targeting the *int* genes of the phages revealed that 21 strains contained one or more prophages (Table 3). Most common were the *int* groups 2 and 6, but groups 1, 7 and 9 were also identified. One t899 strain (09S02250) contained a ΦSa3 prophage and this was the only LA-MRSA CC398 strain which harbored phage-associated virulence genes (*chp*, *sak*). Integration of ΦSa3 phages may also be impeded by a mutated or missing integration site. For that reason, part of the ß-hemolysin gene of the MRSA CC398 strains was amplified by PCR and sequenced. Compared to the published *attB* site for ΦSa3 phages (5′-TGTATCCAAACTGG-3′^33^), all strains revealed two nucleotide exchanges (5′-TGTATCCGAATTGG-3′). To determine whether the endogenous prophages or the nucleotide variations within *attB* may affect lysogenization, all strains were infected with P282 and subsequently analysed. Fourteen out of 32 LA-MRSA CC398 strains (44%) were lysogenized by P282 (Table 3). There was no correlation detectable between lysogenization and the presence of inherent prophages. We then determined the integrity of the lysogens’ ß-hemolysin gene by PCR using primers flanking the *attB* site (see Methods). Between one and five lysogenic colonies of each strain were examined. All colonies of nine strains exhibited an uninterrupted ß-hemolysin gene (Table 3). By contrast, in none of four lysogenic colonies of another strain, a PCR product was detected. Finally, three strains showed only a very weak PCR product. The data indicate that phage P282 may integrate within or outside the ß-hemolysin gene. To determine the alternative P282 integration sites in the *S. aureus* chromosome, DNAs of twelve lysogenic colonies isolated from seven strains were subjected to an outward PCR using several restriction endonucleases (EcoRI, HindIII and XbaI) and primers deduced from the phage integrase gene, followed by sequencing of the amplicons. Altogether, nine alternative integration sites were identified in the strains, which were verified by a control PCR using an *int* primer and a primer deduced from the respective staphylococcal sequence. All phage integrations occurred within genes, most of them encoding metabolic enzymes (Table 4). Four strains revealed two integration sites, while only one site each was detected in the remaining three strains. The sites exhibited identity or strong similarities (maximal two nucleotide exchanges) to the reported *attB* site for ΦSa3 phages. Thus several *attB* related sites exist in LA-MRSA CC398 strains. This was confirmed by analysis of published CC398 whole genome sequences (NC_017333.1, NC_01808.1, NC_017673.1), in which up to three related sites were detected in each strain. At these sites, however, prophages were not identified.

**Table 3:** Lysogenization of livestock-associated CC398 strains by P282.

Strain	*spa* type	Origin	Endogenous prophages (*int* group)	Number of P282 lysogenic colonies	Integrase groups of lysogens	*hlb* intact in P282 lysogenic derivate
07S00067	t5675	swine (nasal swab)	−	−	−	n.d.
07S00107	t2346	swine (nasal swab)	Sa2	2	n.d.	n.d.
08S00699	t2997	swine (dust sample)	Sa1, (Sa5), Sa6	−	−	n.d.
09S00826	t5524	chicken (meat)	Sa2	−	−	n.d.
09S01005	t2346	cattle (nasal swab)	−	−	−	n.d.
09S01007	t2346	cattle (nasal swab)	−	−	−	n.d.
09S01009	t2346	cattle (nasal swab)	−	−	−	n.d.
09S01382	t034	turkey (meat)	Sa2	−	−	n.d.
09S01404	t5902	turkey (meat)	Sa6	−	−	n.d.
09S02250	t899	turkey (skin swab)	Sa3	−	−	n.d.
09S02482	t6574	turkey (meat)	Sa2	3	Sa2, Sa3	+
10S00170	t034	turkey (meat)	Sa6, Sa7	5	Sa6, Sa3	+
10S01355	t034	swine (nasal swab)	−	2	Sa3	+
11S00742	t4677	swine (nasal swab)	Sa6, (Sa7)	−	−	n.d.
11S01169	t2346	cattle (nasal swab)	−	−	−	n.d.
11S01203	t1580	chicken (skin swab)	Sa2, Sa6	5	Sa2, Sa6, Sa3	+
11S01539	t1255	swine (dust sample)	−	1	Sa3	+
12S00070	t2582	chicken (skin swab)	Sa6	1	Sa6, Sa3	+
12S00148	t2123	cattle (dust sample)	Sa1, Sa2	1	Sa1, Sa2, Sa3	(+)
12S00502	t2576	turkey (dust sample)	Sa2	−	−	n.d.
12S00563	t2346	cattle (nasal swab)	−	−	−	n.d.
12S01090	t2346	cattle (nasal swab)	−	−	−	n.d.
12S01091	t2346	cattle (nasal swab)	−	−	−	n.d.
13-ST00013	t034	minced meat	Sa6	4	Sa6, Sa3	−
13-ST00015	t034	turkey (dust sample)	Sa1, Sa6	2	Sa1, Sa6, Sa3	+
13-ST00024	t011	cattle (nasal swab)	Sa2	−	−	n.d.
13-ST00029	t034	cattle (nasal swab)	Sa6	2	Sa6, Sa3	+
13-ST00030	t2346	cattle (nasal swab)	−	−	−	n.d.
13-ST00043	t034	swine (nasal swab)	Sa1, Sa6	−	−	n.d.
13-ST00054	t011	turkey (dust sample)	Sa1, Sa2	1	Sa1, Sa2, Sa3	(+)
13-ST00090	t034	turkey (skin swab)	Sa2, Sa6, Sa9	5	Sa2, Sa6, Sa9, Sa3	+
13-ST00207	t1456	cattle (nasal swab)	Sa2	3	Sa2, Sa3	(+)/−

On the left side all strains lysed by the phage are listed. *Int* groups of prophages residing in the strains are stated. The right side shows numbers of isolated colonies containing P282, their prophage content (*int* groups) and data on the integration of the phage within *hlb*. (+), weak PCR product; n.d., not determined.

**Table 4:** Alternative integration sites of phage P282 in livestock-associated CC398 strains.

Lysogenic isolate	*S. aureus* strain	Attachment site attB*	Integration site (gene)	Reference genome	Accession number
Sa-L35	10-355	TGTATCCAAACTGG	General stress protein (AUC50_04635)	RIVM3897	CP013621
Sa-L36	10-355	TGTATCCAAACTGG	Integrase	Sa54	KT253891
Sa-L48	10-170	TGTATCC**TT**ACTG**T**	Nucleoside permease (AUC50_03495)	RIVM3897	CP013621
Sa-L49	10-170	TGTATCCAA**T**CTGG	General stress protein (AUC50_04635)	RIVM3897	CP013621
Sa-L54	12-070	TGTATCCAAACTGG	Cytochrome D ubiquinol oxidase subunit I (AUC50_05470)	RIVM3897	CP013621
Sa-L56	13-090	TGTATCCAAACTG**A**	Hypothetical protein (AUC50_10740)	RIVM3897	CP013621
Sa-L57	13-090	TGTATCCAAACTG**A**	GMP synthetase (AUC50_02200)	RIVM3897	CP013621
Sa-L67	13-015	TGTATCCAAACTGG	Hypothetical protein (SAMI_1999)	*S. aureus*	AP017320
Sa-L68	13-015	TGTATCCAA**G**CTGG	Gamma-aminobutyrate permease (AUC50_08995)	RIVM3897	CP013621
Sa-L66	13-029	TGTATCCAAACTGG	Gamma-aminobutyrate permease (AUC50_08995)	RIVM3897	CP013621
Sa-L65	13-029	TGTATCCAAACTGG	Gamma-aminobutyrate permease (AUC50_08995)	RIVM3897	CP013621
Sa-L46	11-1203	TGTATCCA**T**ACTGG	Alanine racemase (A7327_11665)	08–02300	CP015646
*attB* within *hlb* P282 *attP*	TGTATCC**G**AA**T**TGG TGTATCC**G**AA**T**TGG				

^*^Nucleotides diverging from the published *attB* integration site for ΦSa3 phages (Coleman *et al*.^33^) are indicated.

### The prophages are stably inherited in MRSA CC398 and their virulence genes are expressed

Previous experiments demonstrated that LA-MRSA CC398 strains can be lysed and lysogenized by virulence gene-containing phages. As prophages might be easily lost, we determined their stability *in vitro*. The lysogenized MRSA CC398 isolates were cultivated in 5 ml LB broth over a period of 15 days by daily inoculation (5 µl) of fresh medium. Every five days, 50 µl aliquots of the cultures were taken and analysed by PCR for the presence of the virulence genes of the prophages. Until the end of the experiment the prophages were detected in all cultures. This does of course not mean that the respective virulence genes are expressed in the MRSA CC398 lysogens. We therefore studied the production of the enterotoxin A, PVL toxin and staphylokinase in the natural hosts of the phages, in LA-MRSA CC398 indicator strains and in the lysogenic derivatives of these strains using a qualitative multiplex antibody microarray^34^. As shown in Table 5 the respective virulence factors were not synthesized in the phage indicator strains, but in both the natural hosts of the phages and in the lysogenized MRSA CC398 derivatives. The detection limits for PVL, SEA and SAK using this assay are 0.5 ng/ml, 0.01 ng/ml and 0.05 ng/ml, respectively^34^. Therefore, the proteins of the investigated strains were produced at least in these quantities. This clearly demonstrates that lysogenization of LA-MRSA CC398 by those phages is accompanied by the production of new virulence factors.

**Table 5:** Virulence factors produced in the original phage host strains, CC398 strains and their lysogenized derivatives.

Strain		PVL^1^	SEA	SAK
11S00282	donor	−	−	+
10S01355	recipient	−	−	−
1355[P282]	lysogenized	−	−	+
11S01105	donor	+	+	−
10S00170	recipient	−	−	−
170[P1105]	lysogenized	+	+	−
13-ST00240	donor	+	−	+
13-ST00030	recipient	−	−	−
30[P240]	lysogenized	+	−	−
10S00630	donor	n.d.	+^2^	n.d.
10S01355	recipient	−	−	−
1355[P630]	lysogenized	n.d.	+^2^	n.d.

^1^lukF-PV/lukS-PV.

^2^Enterotoxin production of these strains was tested with the VIDAS SET 2 kit (bioMérieux, Nürtingen, Germany).

## Discussion

Temperate phages play an important role in the virulence of *S. aureus* and in the emergence of new pathotypes. While prophages containing virulence genes are present in many MRSA strains, they have only rarely been detected in the clonal complex CC398 associated with livestock. The underlying reasons for this finding remain largely unclear. For a ß-hemolysin-converting ΦSa3 phage it has been reported that the phage was not stable in a LA-MRSA CC398 strain^18^. In addition, the ΦSa3 prophage was defective and required a helper phage for infection. McCarthy *et al*.^35^ investigated horizontal gene transfer of phages and plasmids in pigs and did not find any lysogenization of a LA-MRSA CC398 strain by a ΦSa3 phage residing in a human-specific isolate. These data raise the question whether LA-MRSA CC398 strains are suitable hosts for virulence gene-containing phages. To answer this question, we studied infection of LA-MRSA CC398 strains by four temperate phages possessing different combinations of virulence genes. Two phages (P240 and P1105) recovered from human isolates carry genes for the PVL toxin. P1105 additionally contains a *sea* gene for the enterotoxin A. The occurrence of *pvl* and *sea* on the same phage genome is rather uncommon and this combination of virulence genes has to date only been identified in phages isolated in the Far East and in Italy^25, 26^. Notably, all yet described phages harboring *pvl* and *sea* were isolated from ST772 MRSA strains. In contrast, the natural host of PVL phage P240 is a human MRSA CC398 isolate. Both P240 and P1105 are members of *int* group 2 but the phages exhibit only partial homologies. On the other hand, P1105 is closely related to the phages P282 and P630 carrying the genes *chp*, *sak*, *scn* and *sea*, respectively. While IEC genes are widespread in ΦSa3 phages like P282 that occasionally exist in LA-MRSA CC398 strains, *sea* is usually not found in *int* group 6 to which P630 belongs^11^. The content of virulence genes and the relationship of the isolated phages indicate that gene exchange between *S. aureus* phages also occurs across borders of integrase groups. This particularly applies to the DNA region harboring genes for cell lysis, virulence factors and phage integration but probably also to further-upstream sequences. Here, numerous repetitive DNA sequences are located, which are probably implicated in genomic rearrangements. Indeed, recombination between an *int* 2 and an *int* 6 phage has already been detected in pigs^35^.

Plaque assays with the virulence gene-carrying phages clearly showed that a significant number of LA-MRSA CC398 strains are susceptible to these phages. Interestingly, also lysogenic strains containing prophages belonging to the *int* groups 2 and 6 were lysed by P240 and P630, respectively. This shows that LA-MRSA CC398 strains do not lack phage receptors or possess defence systems that could prevent infection. Moreover, endogenous prophages, at least those of the *int* groups 2 and 6 obviously do not confer immunity against phages of the same *int* group indicating that the affiliation to a certain *int* group does not coincide with the specificity of the respective prophage repressor. Thus, also phages belonging to the same *int* group can encounter and recombine in *S. aureus* which may result in new combinations of virulence genes.

All isolated phages were able to lysogenize LA-MRSA CC398 and we did not find any indication for an instability of the prophages. Moreover, no helper phage was needed for lysogenization. However, our experiments were performed *in vitro* and the situation might be different in livestock. It might e.g. be possible that in animals an intact ß-hemolysin gene is required for efficient colonization. Studies with equine and porcine erythrocytes did not support this assumption^36^. Compared to the published 14 bp *attB* attachment site for ΦSa3 phages^37^, all analysed LA-MRSA CC398 strains exhibited two nucleotide exchanges in *hlb* but this alteration did not prevent lysogenization, since integration of P282 within this gene was observed. Furthermore, some LA-MRSA CC398 strains used in our study contain additional integration sites for this phage that are very similar or even identical to the published sequence. Hence, lysogenization by a ΦSa3 phage is not necessarily accompanied by the loss of ß-hemolysin activity. Atypical integration sites for ΦSa3 phages have yet only been reported for disease-related *S. aureus* isolates^38^. They differ from the integration sites that we identified in LA-MRSA CC398 strains.

The other phages of this study are members of the *int* groups 2 and 6. Prophages belonging to these groups are frequently present in LA-MRSA CC398 strains^19^. As shown above they do not protect the host against lytic infection by phages of the same group but they might alleviate the potential of new phages to lysogenize the bacteria because the integration site on the chromosome is already occupied. This fact may explain the rare occurrence of PVL phages belonging to *int* group 2 in LA-MRSA CC398 strains. On the other hand, integration of an *int* group 2 phage at a novel site has recently been reported for a human-specific MRSA CC398 isolate^35^, but corresponding data on livestock-associated strains are lacking. It is still obscure, why virulence gene-containing phages are not common in LA-MRSA CC398. Our results suggest that the natural conditions for lysogenization are fulfilled. Moreover, positive lysogenic conversion of the lysogenized strains was observed. Whether the newly acquired virulence factors are beneficial for MRSA CC398 strains in animals has still to be elucidated. In this study many different CC398 *spa* types were lysed by the phages, most of them are only infrequently found in livestock. By contrast, only some of the predominant t011 and t034 strains were infected. This might be a reason for the rare occurrence of phage encoded virulence factors in livestock. However, it would be naïve to believe that temperate phages do not have the potential to enhance the virulence of those strains. It is probably only a matter of time before livestock-associated strains with new virulence properties may emerge. Indeed, a new subpopulation of CC398 strains isolated from infected animals has recently been described^39^. The strains contained various virulence genes detected by high-density DNA microarray experiments. It is yet not known whether the virulence factors are encoded by prophages, which obtained the respective genes by recombination. It is also not clear what else differentiates these isolates from strains colonizing asymptomatic pigs. Nonetheless, the finding suggests that virulence genes may be spread by phages in CC398 populations resulting in new and possibly more virulent pathotypes posing an inceased risk to human health.

## Methods

### Staphylococcus aureus strains

All strains used in this study were selected from the strain collection of the National Reference Laboratory for Coagulase-Positive Staphylococci incl. *Staphylococcus aureus* at the Federal Institute for Risk Assessment. The strains 11S01105 (original no. 040-07765) and 13-ST00240 (original no. 11-02211) were provided by Robin Köck (University Hospital Münster, Institute of Medical Microbiology, Münster, Germany) and Wolfgang Witte (Robert Koch Institute, Wernigerode, Germany), respectively. MRSA CC398 strains used for the host range determination of the phages are listed in Supplementary Material Table S1. Bacteria were cultivated in lysogeny broth (LB) at 37 °C according to standard procedures (Sambrook and Russel, 2001).

### Microarrays

DNA microarrays were performed using the StaphyType kit (Alere Technologies GmbH, Jena, Germany) according to the manufacturer’s instructions. It covers 333 target sequences including species specific controls, relevant virulence- and resistance genes as well as SCC*mec-*, *agr* group- and capsule typing markers.

Antibody microarrays for the detection of enterotoxin A, staphylokinase and PVL were performed by Alere Technologies GmbH (Jena, Germany) as described by Stieber *et al*.^34^.

### Isolation, propagation and purification of the phages

Phages were released from *S. aureus* strains by mitomycin C induction (2.5 µg/ml). Lysates were centrifuged at 8,000 × g for 15 min and the supernatants were passed through 0.45 µm and 0.2 µm membrane filters (VWR International GmbH, Darmstadt). Lytic activity of the phages was tested by spotting 1:10 dilution series of each lysate onto a lawn of MRSA CC398 indicator strains. Phages were isolated by twofold recovery of single plaques. High titres of phages were achieved by harvesting overlay agar with confluent lysis. The agar was resuspended in SM buffer (100 mM NaCl, 8 mM MgSO_4_ 7H_2_O, 50 mM Tris-HCl, pH 7, 5) and stirred for several hours at room temperature. Thereafter, agar and cell debris were removed by centrifugation and filtration as described above. Phages were concentrated by ultracentrifugation and purified using CsCl step gradients (1,35–1,65 g/cm^2^). To determine phage titers, the softagar overlay method was applied^40^.

### Transmission electron microscopy (TEM)

Phages were applied to pioloform-carbon-coated, 400-mesh copper grids (Plano GmbH, Wetzlar, Germany), for 7 min. Thereafter, grids were fixed with 2.5% aqueous glutaraldehyde solution (w/v) for 1 min, stained with 2% aqueous uranyl acetate solution (w/v) for 1 min and examined by transmission electron microscopy using a JEM-1010 (JOEL, Japan) at 80 kV accelerated voltage.

### Determination of the phage host range

Lytic activity assays were used to determine the host range of the phages. 5 ml of prewarmed LB softagar (0.6%) were mixed with 100 µl of the indicator strain and poured onto a LB agar plate. Following this 10 µl aliquots of 1:10 serial dilutions of each lysate were spotted onto the overlay agar and the plates were incubated overnight at 37 °C. Phage activity was tested on 72 MRSA CC398 strains (Supplementary Table S1).

### Lysogenisation experiments

Isolation of lysogenized MRSA CC398 strains was achieved by removing some softagar from a lysis zone on agar plates (see above). The material was suspended in 500 µl SM buffer and 50–100 µl were plated on LB agar plates and incubated overnight at 37 °C. Colonies were picked and investigated by PCR for the presence of phage encoded virulence genes.

### PCR analyses

Bacterial DNA was extracted by thermal lysis. A 1 µl loop of culture material was suspended in 45 µl TE buffer (10 mM Tris-HCl, 1 mM EDTA) and 5 µl lysostaphin (0.1 mg/µl). After incubation for 45 min at 37 °C, the sample was heated at 95 °C for 10 min and cooled by adding 200 µl TE buffer.

For the detection of virulence genes, the following primers were used: (*sak*-F, 5′-GTAAGTGCATCAAGTTCATTCG-3′; *sak*-R, 5′-GCTCTGATAAATCTGGGACAAC-3′; *scn*-F, 5′-AATGGCTCTTCTTCGCTTTC-3′; *scn*-R, 5′-TGCTACTTTATTCCTTACGGC-3′; *chp*-F, 5′-GTTTTTTAACGGCAGGAATCAG-3′; *chp*-R, 5′-TTTCTATCTTCAGCAAGTGGTG-3′), (*hlb*1B, 5′-GTTGCAACACTTGCATTAGCA-3′; *hlb*2, 5′-TGTGTACCGATAACGTGAAC-3′); (*pvl*-F, 5′-TTGAAATGTTGTACTTAGAACC-3′; *pvl*-R, 5′-TAGGTAAAATGTCTGGACATG-3′). Primers used for the detection of the enterotoxin A gene *sea* and phage integrase genes have previously been described by Johnson *et al*.^41^, Goerke *et al*.^11^ and Kahankova *et al*.^42^. PCR was performed using 2x MyTaq HS Mix (Bioline, Luckenwalde, Germany) with a final volume of 25 µl containing 10 µl MyTaq HS Mix, 1 µM of forward and reverse primer, 1 µl DNA (10 ng) and dH_2_O.

For the determination of the *attB* site of ΦSa3 phage P282 within *hlb*, PCR products of the β-hemolysin gene were purified using the Invisorb Spin DNA Extraction Kit (Stratec Molecular, Berlin, Germany) and sequenced (Eurofins Genomics, Ebersberg, Germany).

### Determination of alternative phage P282 integration sites

For the determination of the chromosomal integration sites (*attB*) of P282, genomic DNA (gDNA) of various lysogens was isolated using the DNA mini preparation kit (Qiagen, Hilden, Germany). DNAs were digested with the restriction endonucleases Eco32I, HindIII, and XbaI according to the manufacturer’s recommendations (Thermo, St. Leon Roth, Germany). Following digestion, restriction fragments were treated with T4 ligase (Thermo) and used as template for PCR. Outward primers deduced from the 5′- and 3′-coding region of the P282 *int* gene were used under the following conditions: Initial PCR activation and template denaturation at 96 °C for 120 s followed by 35 cycles including denaturation phase at 96 °C for 15 s, annealing at 55 °C for 5 min and elongation for 210 s at 72 °C. In addition, a final elongation step at 72 °C for 1 min was included, before the amplicons were purified (QIAquick PCR purification kit, Qiagen) and sequenced according to Sanger (Eurofins Genomics,). The obtained nucleotide sequences of the amplicons were compared with whole genome sequences of *S. aureus* strains of the GenBank database (National Center for Biotechnology Information).

### Extraction of phage DNA, sequencing and bioinformatics analyses

Phage DNA was extracted by incubating CsCl-purified phage particles (400 μl) in 10 mM Tris-HCl (pH 7.5), 1 mM EDTA, 0.5% SDS supplemented with 2 μl Proteinase K (20 mg/ml) at 56 °C for 2 h, followed by ethanol precipitation^40^.

Sequencing of the phages P282 and P630 was performed using the PacBio RS system by GATC Biotech (Konstanz, Germany). *De novo* genome assemblies of raw reads (P282: 37,713; P630: 39,145) of one SMRT cell (run modus: 1 × 180 min movie) were obtained by SMRT analysis (version 2.3.0) software (Pacific Biosciences, USA) resulting in a sequence coverage of >100-fold per consensus base for both phage genomes. HGAP3-assembling resulted in a single sequence contig of 41,960 bp and 40,448 bp for phage P282 and P630, respectively.

To determine the P240 and P1105 genomic sequences, EcoRI and HindIII restriction fragments of the phages were inserted into the linearized vector pIV2^43^. *Escherichia coli* strain GeneHogs (Invitrogen, Carlsbad, USA) was electroporated with aliquots of the respective ligation mixtures. Transformants were selected on LB agar supplemented with neomycin (100 µg/ml), X-Gal and IPTG^40^. Following isolation of recombinant plasmids (Plasmid Mini Two Kit, Stratec, Berlin, Germany) inserts were sequenced (Eurofins Genomics) using vector primers^43^. Based on the strong homologies to known phages, the whole genomic sequences of P240 and P1105 were determined by PCR and direct sequencing of phage DNA using primers deduced from the phages SMSAP5 (NC_019513.1) and PhiIND772PVL^25^.

Sequence analysis and alignments were carried out using Accelrys Gene (version 2.5, Accelrys Inc., San Diego, CA, USA). ORF analyses were carried out using MyRAST^44^. Similarity and identity values were determined at the NCBI homepage using different BLAST algorithms^30^. Transcription terminators were identified using Arnold^45^.

### Nucleotide sequence accession number

The complete nucleotide sequence of the phage genomes were submitted to GenBank under the accession numbers KT809368 (P282), KT809369 (P630), KT878766 (P1105) and KY056620 (P240)﻿P240.

### Originality-Significance Statement

In this work the potential of temperate phages to increase the virulence of livestock-associated MRSA CC398 strains has been elucidated. We demonstrate that many CC398 strains can be lysogenized by virulence gene containing phages, that lysogeny is stable and that the respective virulence factors are synthesized. In addition, we show that integration of a ΦSa3 phage may occur at numerous sites on the CC398 chromosome.

## Electronic supplementary material

Figures S1 and S2, Table S1

Table S2
