# Supplementary material for: Acquisition of virulence factors in livestock-associated MRSA: Lysogenic conversion of CC398 strains by virulence gene-containing phages
Source: Sci Rep. 2017 May 17;7:2004. doi: 10.1038/s41598-017-02175-4 (PMC5435737; doi:10.1038/s41598-017-02175-4)
Supplement: Supplementary file 1 — Figures S1 and S2, Table S1 [file 41598_2017_2175_MOESM1_ESM.pdf]

Acquisition of virulence factors in livestock-associated MRSA: Lysogenic conversion of CC398 strains by virulence gene-containing phages

Britta Kraushaar[1], Jens Andre Hammerl[1], Marina Kienöl[1], Marie Luise Heinig[1], Nina Sperling[1], Mai Dinh Thanh[1], Jochen Reetz[1], Claudia Jäckel[1], Alexandra Fetsch[1] and Stefan Hertwig[1,*]

Supplemental Material

Supplemental Material Figure S1: DNA similarities of the four phages. Dot plot alignments were performed with the DS Gene software package (v 2.5) of the Accelrys Inc. (USA) by using 65% as a standard parameter for DNA similarity and a hash value of 6. The numbers on the axes give the scale for the genomes in kilo bases.

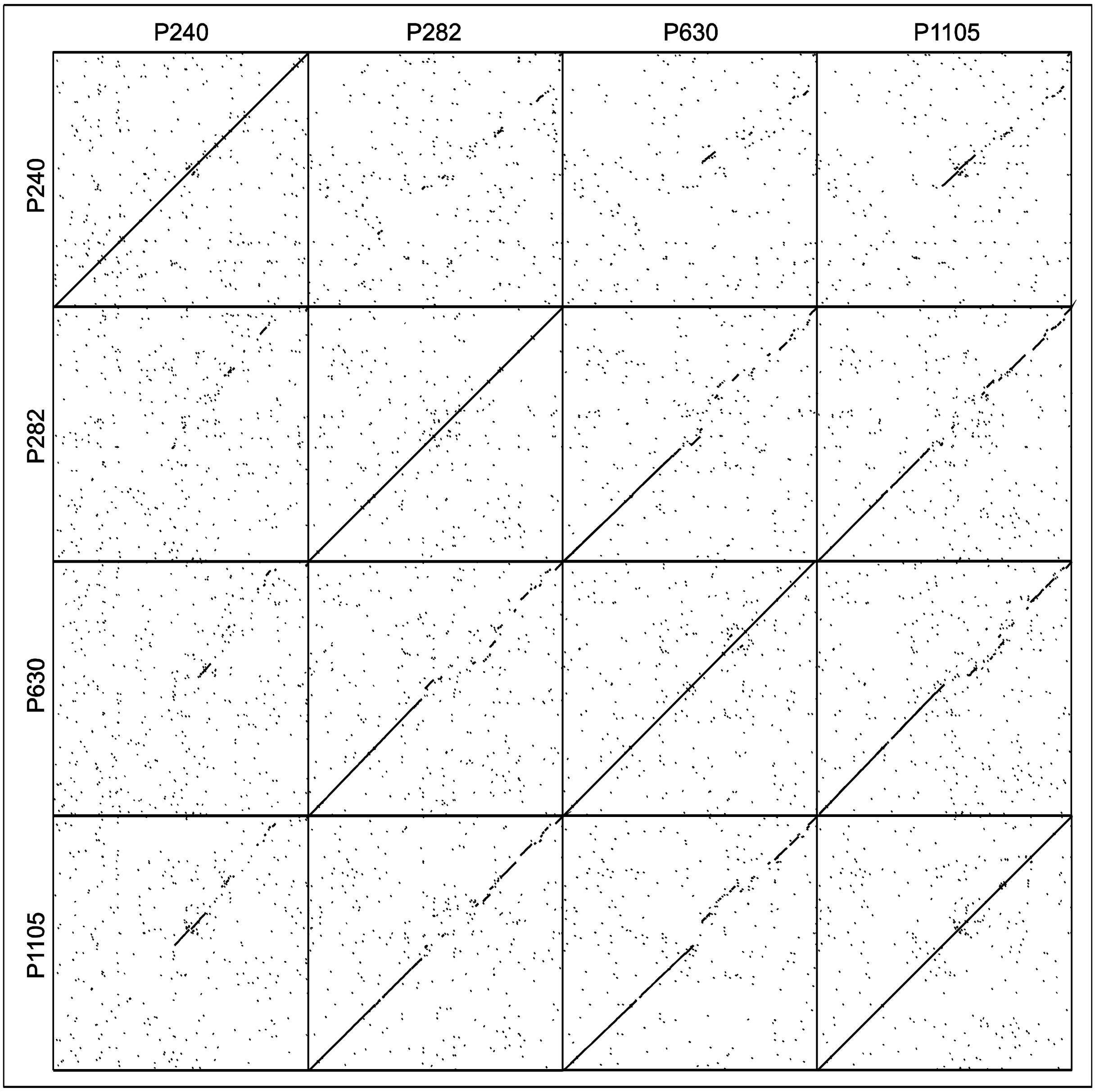

Supplemental Material Figure S2: Similarities of P1105 to other related phages. Dot plot alignments were performed with the DS Gene software package of the Accelrys Inc. (USA) by using 65% as a standard parameter for DNA similarity and a hash value of 6. The numbers on the axes give the scale for the genomes in kilo bases. The transition region between homologous and non-homologous DNA sequences is framed by a red box.

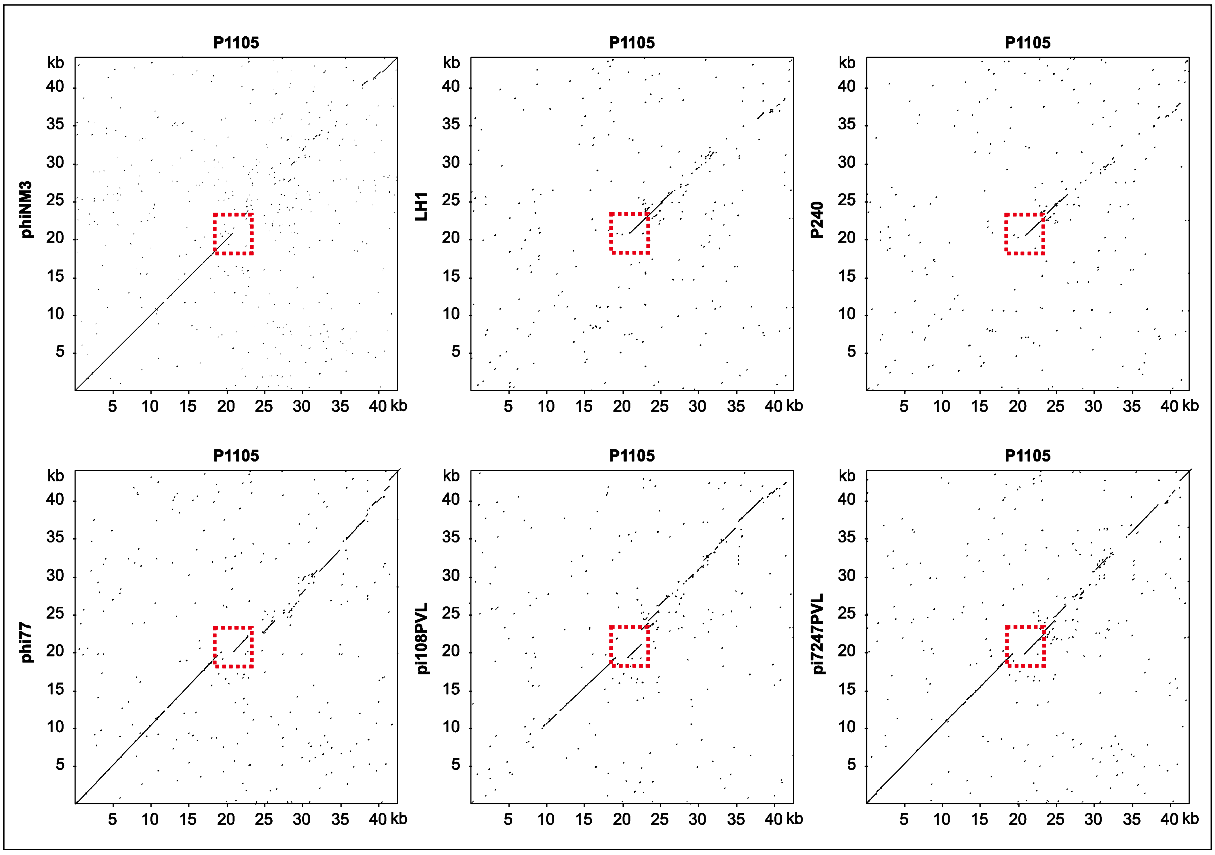

Supplemental Material Table S1: Livestock-associated CC398 strains used to determine the host range of the phages.

Supplemental Material

Supplemental Material Figure S1: DNA similarities of the four phages. Dot plot alignments were performed with the DS Gene software package (v 2.5) of the Accelrys Inc. (USA) by using 65% as a standard parameter for DNA similarity and a hash value of 6. The numbers on the axes give the scale for the genomes in kilo bases.

Supplemental Material Figure S2: Similarities of P1105 to other related phages. Dot plot alignments were performed with the DS Gene software package of the Accelrys Inc. (USA) by using 65% as a standard parameter for DNA similarity and a hash value of 6. The numbers on the axes give the scale for the genomes in kilo bases. The transition region between homologous and non-homologous DNA sequences is framed by a red box.

Supplemental Material Table S1: Livestock-associated CC398 strains used to determine the host range of the phages.

**Strain**	***spa* type**	**Origin**	**P282**	**P630**	**P1105**	**P240**
07S00067	t5675	swine (nasal swab)	+	-	-	-
07S00107	t2346	swine (nasal swab)	+	-	-	+
07S00108	t5681	swine (nasal swab)	-	-	-	-
07S00289	t2346	swine (nasal swab)	-	-	-	-
07S00338	t5676	swine (nasal swab)	-	-	+	-
07S00396	t5680	swine (nasal swab)	-	-	-	-
08S00699	t2997	swine (dust sample)	+	-	-	-
08S00915	t3423	swine (meat)	-	+	-	-
08S01074	t2346	swine (nasal swab)	-	-	-	-
08S01077	t2346	swine (nasal swab)	-	-	-	-
08S01578	t3479	swine (nasal swab)	-	-	-	-
09S00826	t5524	chicken (meat)	+	-	-	+
09S01005	t2346	cattle (nasal swab)	+	-	-	-
09S01007	t2346	cattle (nasal swab)	+	-	+	+
09S01009	t2346	cattle (nasal swab)	+	+	+	+
09S01255	t2330	turkey (tracheal swab)	-	-	-	-
09S01385	t2346	turkey (meat)	+	-	-	+
09S01404	t5902	turkey (meat)	+	+	-	+
09S01590	t5119	turkey (meat)	-	-	-	-
09S01727	t026	swine (meat)	-	-	-	-
09S02250	t899	turkey (skin swab)	+	-	-	+
09S02459	t6575	swine (organs)	-	-	-	-
09S02482	t6574	turkey (meat)	+	+	-	+
09S02531	t2971	swine (meat)	-	-	-	-
10S00170	t034	turkey (meat)	+	+	+	-
10S01160	t2970	turkey (skin swab)	-	-	-	-
10S01468	t2370	minced meat	-	-	-	-
10S01531	t2510	swine (dust sample)	-	-	-	-
10S01600	t779	swine (dust sample)	-	-	-	-
11S00157	t2510	swine (dust sample)	-	-	-	-

11S00724	t588	swine (nasal swab)	-	-	-	-
11S00742	t4677	swine (nasal swab)	+	-	-	-
11S01169	t2346	cattle (nasal swab)	+	-	+	+
11S01203	t1580	chicken (dust sample)	+	+	-	+
11S01539	t1255	swine (dust sample)	+	+	+	+
12S00070	t2582	chicken (skin swab)	+	+	-	-
12S00148	t2123	cattle (dust sample)	+	-	-	+
12S00502	t2576	turkey (dust sample)	+	-	-	-
12S00562	t4652	turkey (skin swab)	-	-	-	-
12S00563	t2346	cattle (nasal swab)	+	-	-	+
12S00746	t1344	turkey (skin swab)	-	-	-	-
12S00887	t1928	turkey (dust sample)	-	-	-	-
12S01090	t2346	cattle (nasal swab)	+	-	-	-
12S01091	t2346	cattle (carcass)	+	-	-	-
12S01431	t5452	turkey (dust sample)	-	-	-	-
12S01487	t2346	swine (nasal swab)	-	-	-	-
13-ST00001	t034	turkey (dust sample)	-	-	-	-
13-ST00003	t034	swine (boot swab)	-	-	-	-
13-ST00007	t1451	swine (nasal swab)	-	-	-	-
13-ST00008	t108	swine (nasal swab)	-	-	-	-
13-ST00009	t034	cattle (carcass)	-	-	-	-
13-ST00010	t1197	cattle (carcass)	-	-	-	-
13-ST00013	t034	minced meat	+	-	-	-
13-ST00015	t034	turkey (dust sample)	+	-	-	-
13-ST00024	t011	cattle (nasal swab)	+	-	-	-
13-ST00026	t011	cattle (nasal swab)	-	-	-	-
13-ST00027	t6325	cattle (carcass)	-	-	-	-
13-ST00029	t034	cattle (carcass)	+	+	-	-
13-ST00030	t2346	cattle (carcass)	+	-	+	+
13-ST00042	t108	swine (nasal swab)	-	-	-	-
13-ST00043	t034	swine (nasal swab)	+	-	-	-
13-ST00053	t034	turkey (dust sample)	-	-	-	-
13-ST00054	t011	turkey (dust sample)	+	-	-	+

13-ST00065	t1197	cattle (carcass)	-	-	-	-
13-ST00089	t5452	turkey (skin swab)	-	-	-	-
13-ST00090	t034	turkey (skin swab)	+	+	-	-
13-ST00093	t011	cattle (carcass)	-	-	-	-
13-ST00117	t2876	turkey (skin swab)	-	-	-	-
13-ST00193	t216	rabbit (organs)	-	-	-	-
13-ST00195	t011	chicken (meat)	-	-	-	+
13-ST00207	t1456	cattle (nasal swab)	+	-	-	+
13-ST00385	t571	cattle (nasal swab)	-	-	-	-
